# Supplementary material for: In vitro and in vivo comparison of transport media for detecting nasopharyngeal carriage of Streptococcus pneumoniae
Source: PeerJ. 2016 Sep 22;4:e2449. doi: 10.7717/peerj.2449 (PMC5036082; doi:10.7717/peerj.2449)
Supplement: Supplemental Information 3 — This file shown the method and results of the tests we did before the actual in vitro experiment, to determine the detection limits for pneumococcal detection. [file peerj-04-2449-s003.docx]

**Determination of the detection limits for pneumococcal detection**

Online material of “*In vitro* and *in vivo* comparison of transport media for detecting nasopharyngeal carriage of *Streptococcus pneumoniae*”. Steens et al.

Aim

To determine the detection limits for pneumococcal detection by CFU counting, DNA quantification and latex agglutination, which has been used in the actual study presented in the main manuscript.

Methods

We determined the lower detection limits for pneumococcal detection by colony forming units (CFU) counting, DNA quantification and latex agglutination for serotypes 19F (ATCC 49619 reference strain) and serotype 4 (TIGR4). Colonies from each serotype were suspended in Todd-Hewitt (TH) broth at a concentration of 0.5 McFarland in serial 1:10 dilutions, down to a dilution of 10^-9^ McFarland. From each dilution, 100µL was plated on Columbia horse blood agar plates, and 200µL was used for DNA extraction for the calibration curve. All was done in triplicate. Agar plates and TH dilutions were then incubated overnight at 35°C with 5% CO_2_. The following day, colony forming units (CFU) were counted in each plate, and latex agglutination and quantitative real-time PCR (qPCR) were performed for each dilution. See paper for details.

Results

Figure 1 (see below) presents the results determined by qPRC (Ct) and plating (CFU). Recovery of pneumococcal DNA was possible down to 10^-5^ dilution, after which DNA was not detected (Ct>40). Colonies were countable from the interval of dilutions 10-5 to 10-7, above which there were too many colonies to count and below which no colony growth was observed.

Conclusion

For the *in vitro* component of the study, serial 1:10 dilutions of 10^-2^ to 10^-5^ from the initial TH broth at a concentration of 0.5 McFarland will be used as that coveres the minimum detection ranges for both DNA quantification and CFU counting.


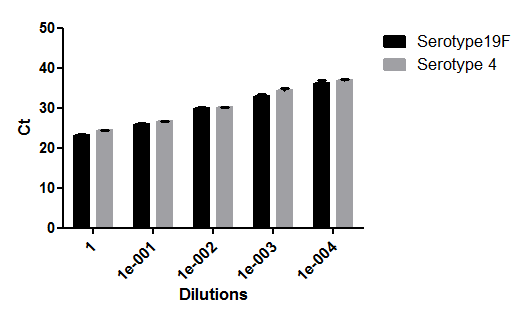

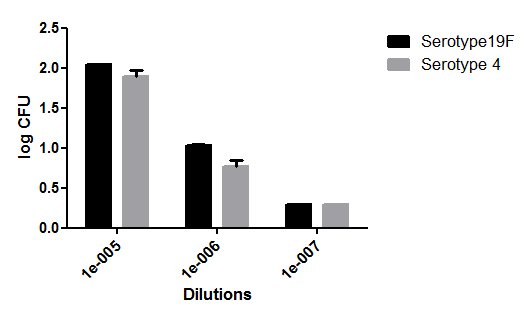


*Figure 1: DNA quantification (Ct values) and log of CFU of serotypes 19F and 4 in Todd-Hewitt medium*
